# Fisheries data management systems in the NW Mediterranean: from data collection to web visualization

**DOI:** 10.1093/database/baad067

**Published:** 2023-10-20

**Authors:** Jordi Ribera-Altimir, Gerard Llorach-Tó, Joan Sala-Coromina, Joan B Company, Eve Galimany

**Affiliations:** Institut de Ciències del Mar (ICM-CSIC), Passeig Marítim de la Barceloneta 37-49, 08003 Barcelona, Catalonia, Spain; Institut Català de Recerca per a la Governança del Mar (ICATMAR), Passeig Marítim de la Barceloneta 37-49, 08003 Barcelona, Catalonia, Spain; Institut de Ciències del Mar (ICM-CSIC), Passeig Marítim de la Barceloneta 37-49, 08003 Barcelona, Catalonia, Spain; Institut Català de Recerca per a la Governança del Mar (ICATMAR), Passeig Marítim de la Barceloneta 37-49, 08003 Barcelona, Catalonia, Spain; Xarxa Marítima de Catalunya (BlueNetCat), Plaça d’Eusebi Güell 6, 08034 Barcelona, Catalonia, Spain; Institut de Ciències del Mar (ICM-CSIC), Passeig Marítim de la Barceloneta 37-49, 08003 Barcelona, Catalonia, Spain; Institut Català de Recerca per a la Governança del Mar (ICATMAR), Passeig Marítim de la Barceloneta 37-49, 08003 Barcelona, Catalonia, Spain; Institut de Ciències del Mar (ICM-CSIC), Passeig Marítim de la Barceloneta 37-49, 08003 Barcelona, Catalonia, Spain; Institut Català de Recerca per a la Governança del Mar (ICATMAR), Passeig Marítim de la Barceloneta 37-49, 08003 Barcelona, Catalonia, Spain; Institut de Ciències del Mar (ICM-CSIC), Passeig Marítim de la Barceloneta 37-49, 08003 Barcelona, Catalonia, Spain; Institut Català de Recerca per a la Governança del Mar (ICATMAR), Passeig Marítim de la Barceloneta 37-49, 08003 Barcelona, Catalonia, Spain

## Abstract

The European Union Data Collection Framework (DCF) states that scientific data-driven assessments are essential to achieve sustainable fisheries. To respond to the DCF call, this study introduces the information systems developed and used by *Institut Català de Recerca per a la Governança del Mar* (ICATMAR), the Catalan Institute of Research for the Governance of the Seas. The information systems include data from a biological monitoring, curation, processing, analysis, publication and web visualization for bottom trawl fisheries. Over the 4 years of collected data (2019–2022), the sampling program developed a dataset of over 1.1 million sampled individuals accounting for 24.6 tons of catch. The sampling data are ingested into a database through a data input website ensuring data management control and quality. The standardized metrics are automatically calculated and the data are published in the web visualizer, combined with fishing landings and Vessel Monitoring System (VMS) records. As the combination of remote sensing data with fisheries monitoring offers new approaches for ecosystem assessment, the collected fisheries data are also visualized in combination with georeferenced seabed habitats from the European Marine Observation and Data Network (EMODnet), climate and sea conditions from Copernicus Monitoring Environment Marine Service (CMEMS) on the web browser. Three public web-based products have been developed in the visualizer: geolocated bottom trawl samplings, biomass distribution per port or season and length-frequency charts per species. These information systems aim to fulfil the gaps in the scientific community, administration and civil society to access high-quality data for fisheries management, following the Findable, Accessible, Interoperable, Reusable (FAIR) principles, enabling scientific knowledge transfer.

**Database URL** https://icatmar.github.io/VISAP/(www.icatmar.cat)

## Introduction

Global fishing stocks have been declining in the last decades because of both overfishing and an increasing demand for fish, which has led to the loss of catches since the 1950s (1). Aquaculture has been proposed to compensate for the lack of fish and increase marine resources to feed the human population. However, aquaculture itself will not be able to overcome the problem of marine food scarcity because it requires great amounts of wild-caught fish as feed (2). Instead, stocks that are scientifically assessed tend to increase in abundance with numbers trending towards proposed target levels (3). Therefore, there is a need to develop effective fisheries management strategies to sustain fisheries and the socio-economics involved.

The European Union Data Collection Framework (DCF) established for member states the obligation to collect, manage and annually report biological, environmental and socioeconomic fisheries data as a source to provide scientific advice for management purposes (EU 2017/1004). In the Mediterranean, fisheries data collection is carried out through both fisheries-dependent data (monitoring of commercial fisheries on board of fishing vessels and in auction), and fisheries-independent data (annual scientific trawl samplings for demersal species and acoustic surveys for small pelagic species). Other initiatives, apart from the official monitoring efforts from the DCF, also collect fisheries data. For example, the *Institut Français de Recherche pour l'Exploitation de la Mer*, the national institute for ocean science in France, implemented a Fisheries Information System (FIS) aiming to monitor their fisheries with scientific goals (4). In the USA, fisheries monitoring programs vary according to geography, objectives, practices, technology, institutional structures and funding (5). This specificity is important and adds value to the data obtained to better implement science-based management strategies. This was the main idea behind the creation of the *Institut Català de Recerca per a la Governança del Mar* (ICATMAR), a research institute devoted to obtain oceanographic data and implement marine monitoring programs with the goal to support marine governance and management in the blue economy field. ICATMAR is an organization embedded within the Institut de Ciències del Mar (ICM-CSIC) and the Generalitat de Catalunya, the autonomous government of Catalonia (NE Spain). Since 2019, ICATMAR has developed and implemented a fisheries’ monitoring program targeting the main commercial species of the Catalan coast using three fishing modalities (bottom trawling, purse seine and small-scale fisheries).

Traditionally, fisheries monitoring data are collected in written logs, analyzed and used to generate reports. However, new emerging technologies and advanced data systems ‘have the potential to contribute to fishery-dependent data systems by expanding or streamlining data collection, automating and empowering the data processing and analysis and facilitating the communication of the results to relevant stakeholders (6). Even though data collection is typically funded by government organizations and public institutions, the majority of fisheries monitoring programs do not provide access to the data through open portals, and the data are often private or not easily accessible. For example, despite that the tracks of the fishing vessels (Vessel Monitoring System, VMS) are used for control purposes, both VMS and fishing landings can be valuable data for research. However, these data are not easily available or accessible because the corresponding institutions need a formal request in order to obtain the data. The reality of the scientific data is, then, opposed to the FAIR (Findable, Accessible, Interoperable, Reusable) principles (7). Effective data management is essential to all potential stakeholders that may use fisheries information. The FAIR principles stress the need to use automated machines to find data and allow their use by a varied group of stakeholders including industry, academia, agencies and scientists (7).

In this work, we present the process of collecting, ingesting, organizing and visualizing the data of the bottom trawl fisheries’ monitoring program from ICATMAR. The data system and visualization aim to easily provide data to different fishing sectors including fishing guilds and administration, the scientific community and the public in general. Data visualization is accessible to all stakeholders through an open website, based on FAIR principles, for the transfer of scientific knowledge, dissemination and education. Bathymetry, seabed habitats and climate and sea conditions are added to the visualization, as they are important factors that influence the catch composition (8–10).

## Materials and methods

### Study area

**Figure 1.**
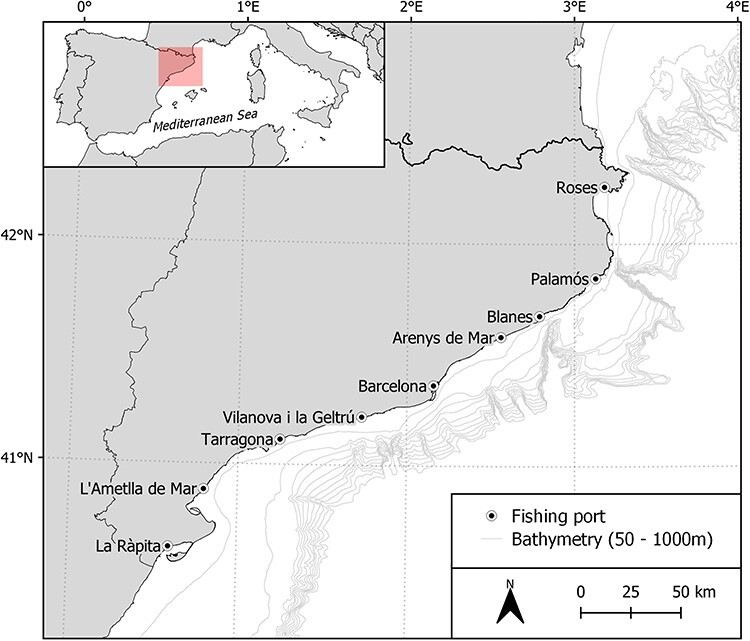
Map showing the study area along the Catalan coast (NW Mediterranean Sea), where the bottom trawling samplings take place). Sampling fishing ports are displayed on the map.

The Catalan coast (NW Mediterranean) is the study area where the data are collected (Figure 1), covering the depths where bottom trawling fishing is permitted. These depths range between 50 and 1000 m with the exception that the minimum depth may be exchanged for a minimum distance of 3 nautical miles from the coast in shallow areas, where the 50 m depth are reached at a farther distance from the coast (EC 1626/1994; EC 1967/2006). This study comprises the bottom trawl depths where demersal resources are mainly exploited, targeting European hake (*Merluccius merluccius*), red mullet (*Mullus barbatus*), horned octopus (*Eledone cirrhosa*) and a variety of crustaceans, i.e. Norway lobster (*Nephrops norvegicus*), blue and red shrimp (*Aristeus antennatus*), spottail mantis shrimp (*Squilla mantis*), deep-water rose shrimp (*Parapenaeus longirostris*) and caramote prawn (*Penaeus kerathurus*). In Catalonia, the greatest fishing revenue is obtained by the bottom trawl fishery as a result of the high price of some target species. In 2021, this fishery landed 6,603.9 t and generated 55.6 million €, which represent 34.8% and 60.8% of the total fishing catch and revenue, respectively (11).

### Data description and collection

**Figure 2.**
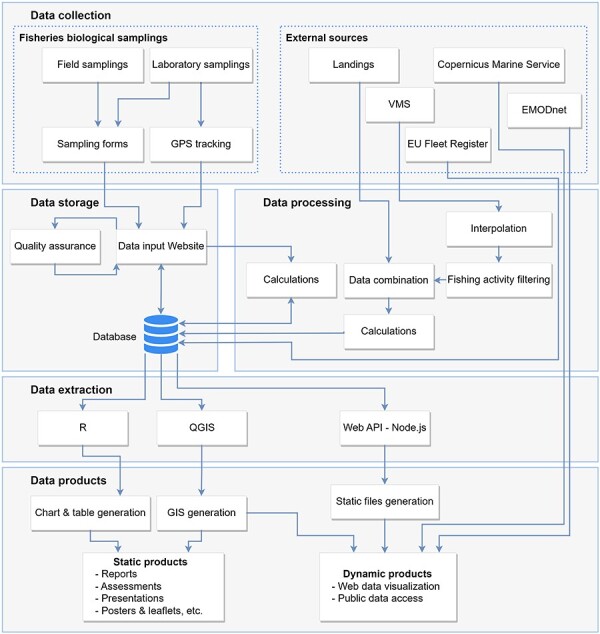
Diagram of the data workflow: data collection, processing, storage, extraction and products.

Scientific trawling data collection began in 2019 and it is still ongoing through onboard samplings and posterior laboratory analysis. External fisheries data of the monitoring program (such as daily commercial fishing landings, VMS and EU fleet register) provided by the diverse administrations and other online sources are also collected for data analyses. ICATMAR’s data management systems allow storage of the collected data in a database, automatic data processing, integration with external sources, data sharing and visualization (Figure 2).

Onboard sampling data contain information from daily onboard catches performed at three different depths, one haul at each depth, following the recommendations described in Blanco *et al.* 2023 (12). For each haul, and briefly, the catch is identified at species level and measured. Then, two different subsamples of the catch are brought to the laboratory: (i) a subsample of the target species is used for biological and reproductive studies; (ii) a subsample of the discarded fraction of the catch, including commercial species under the minimum conservation reference size (MCRS), non-commercial species, natural debris and marine litter. This fraction is identified and weighted, and all individuals are measured. Along with the catch composition analysis, all haul positions are registered using a GPS device with point data collected at a frequency of 1 min. Onboard positions are also manually collected every 15 min from the start to the end of the fishing haul.

The ingestion of onboard and laboratory sampling data is done through an internal data input website. It was developed using Django, an open-source framework based on Python (13). Using the website, the GPS tracks (.gpx files) are uploaded for each haul together with the positions collected manually onboard. Data are automatically transferred from the input website to ICATMAR’s database for storage and further processing. The open-source database used is PostgreSQL (14) with PostGIS extension (15).

The database was designed as a relational database. The model of the database was designed by grouping the related fields in indexed tables according to each data concept, relating them with its corresponding cardinality and seeking data redundancy reduction (Supplementary Figure S1). From the data input website, Django’s ORM (Object-Relational Mapper) is used to interact with the database. This technique creates a virtual object database, which can be accessed via the programming language.

**Figure 3.**
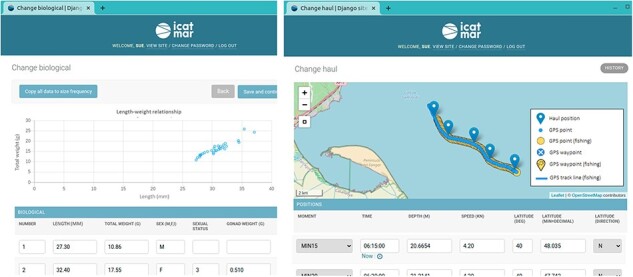
Examples of the data input website displaying interactive charts and maps to perform visual validations of the entered data. The scatter chart displays the individuals’ lengths and weights to detect possible outliers. The interactive chart is displaying the onboard positions (blue pointers), together with the uploaded GPS points (blue circles) and the GPS points detected as fishing (yellow circles).

All data collected go through a validation workflow to ensure high quality in their analysis and publishing. Using the data input website to ingest onboard and laboratory sampling data allows having more control of the data, prevents defects and facilitates validation, data availability and system reliability. This database includes parametrized text fields and the numeric fields with data ranges. Different validations are performed during the data input workflow to trigger warnings and detect errors to prevent defects. Finally, the data are displayed in interactive maps and charts to perform visual validations (Figure 3). The interactive maps use the library Leaflet (16) and the interactive charts use the library Chartjs v2.8 (17). At the end of each season, R scripts are executed to check joint data completeness and perform a general data validation.

There are three main external fisheries data sources that are integrated into ICATMAR’s database and linked between them, as detailed below:

- Daily commercial fishing landings provided by the Catalan government (Department of Climate Action, Food and Rural Agenda) since year 2000. The trawling fishing fleet of Catalonia has a daily fishing time limit of 12 h (Real Decreto 1440/1999) and an obligation to land the catches daily. Then, fish sales take place at the auctions upon the arrival of the vessels. All these data are registered and transmitted to the Catalan government daily, providing information on the landing date, fishing vessel, port, species, weight and income.
- Bottom trawling VMS records provided by the General Fisheries Secretariat of the Spanish Ministry of Agriculture, Food and Environment since year 2008. VMS is a satellite-based monitoring system that provides data at regular intervals, at least once every 2 h, specifying the location, course and speed of each trawling vessel (Reg. EC 2371/2002; Reg. EC 1224/2009).
- EU fleet register provided by the European Commission. The EU fleet register provides extra registration information and technical characteristics about the trawling fleet (Reg. EU 2017/218).

### Data processing for standard metrics

Following the DCF guidelines (Reg. EU 2017/1004 and Reg. EU 2019/910), all the collected data are processed to obtain comparable standardized metrics to enable valid estimates that facilitate the fisheries assessment, such as fishing effort, species length distribution or biomass per area (km^2^).

Geographical information and fishing gear characteristics are needed to calculate the swept area of the onboard samples from the trawling hauls, used to standardize the abundance and biomass of the different species. The Global Positioning System (GPS) positions are automatically selected and cut by the data input website using the onboard positions to determine the start and end of a haul. The resulting GPS track, together with the trawling net width, is used to calculate the swept area.

The length distribution analysis for the fished species provides valuable insight into the populations’ dynamics being a commonly used parameter for the assessment of fisheries. To analyze the length frequencies of each species per each haul, individual sizes are categorized into length classes for each sample (fraction of landed or discarded species). Then, the abundances, expressed as number of individuals per km^2^, for each species (*x*), length class (*y*) and sample (*z*) are calculated and stored in the database following the equation:


(1) $$Abundanc{e_{xyz}}\,\left( {\frac{{Nindividuals}}{{k{m^2}}}} \right) = \frac{{{F_{xyz}}\,*\,S{P_z}\,*\,D{P_z}}}{{SD\,*\,GW}}\,$$

where $F$ is the number of individuals for the species *x,* length class *y* and sample *z* within a haul, *SP* is the proportion of the subsample, *DP* is the proportion of the discard fraction (only used when calculating discards), *SD* is the swept distance of a haul and *GW* is the width of the gear.

Besides obtaining the length distribution for the main commercial species, it is important to analyze the overall composition of the catch to evaluate the fisheries and their exploited fishing grounds. Thus, to analyze the catch composition from a haul, the weight of the debris and density, expressed as kilograms per kilometre squared, for each species (*x*), category (*k*) -landed, discarded, natural debris and marine litter- and sample (*z*) are calculated and stored in the database following the equation:


(2) $$Biomas{s_{xkz}}\left( {\frac{{kg}}{{k{m^2}}}} \right) = \frac{{\mathop \sum \nolimits_{i = 1}^N \left( {{a_x}\,*\,L_i^{{b_x}}} \right)*\,{F_{x{L_i}z}}\,*\,S{P_z}\,*\,D{P_z}\,}}{{SD*GW}}\,$$

where *N* is the number of individuals for the species *x*, category *k* and sample *z* within a haul; and *L* is the length of each individual *i*, which is transformed to weight using the corresponding length–weight relationship of the species *x* (*a* and *b* parameters). The length–weight relationship is only applied to the individuals measured during the field sampling because they are not weighted, as opposed to those sampled in the laboratory where the weight of all individuals is determined. Thus, the weight from the individuals only measured in length is calculated using the length–weight equation established with the laboratory individuals for each species. The rest of the parameters are explained in Equation. 1. As the debris weight is also standardized to kilograms per kilometre squared, from now on both calculations will be referred to as biomass.

**Figure 4.**
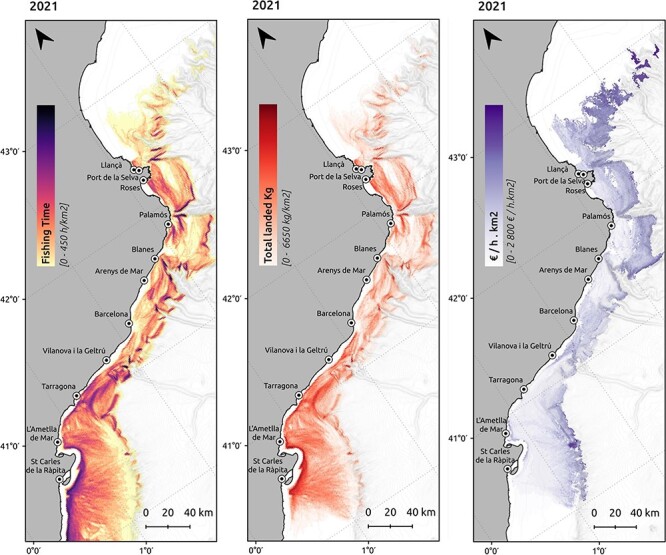
Spatial distribution of the fishing effort (h/km^2^), landings (km/km^2^) and revenues per unit of effort (€/h· km^2^) produced by the bottom trawling fishing fleet in 2021.

The VMS and landings data are combined to obtain fishing effort maps (kilograms landed) and revenue (€ generated) per kilometre squared, for all the Catalan trawling fleet (Figure 4). All VMS landings are processed automatically via functions and triggering the data workflow.

For data processing, the same protocol described in Sala-Coromina *et al.* (18) is applied. In summary, *VMSbase* R package (19) is used to remove duplicate registers and points on land. Then, the point frequency is increased by interpolating points at 10 min resolution. Ping series corresponding to the same vessel tracks are identified with a track code, unique for each fishing trip (day and vessel). The resulting data are introduced in ICATMAR’s database for further analyses. To identify the trawling fishing activity of a vessel, a speed filter is applied to the interpolated VMS data. This filter includes the speed range for fishing and excludes the steaming and inactive moments of the vessel. Moreover, a depth filter is applied to discard erroneous points located out of the fishing zones. Then, the total fishing time (hours) is calculated for each track.

The fishing VMS positions dataset is then combined with the landings dataset. The variables contained in the landings dataset are weight and revenue per species and fishing trip (day and vessel); therefore, they can be linked to the VMS data through a shared codification of fishing trips (day and vessel). That is, total fishing time, landings and revenues by species of a fishing trip are distributed equally among all fishing positions for the same day and vessel. As a result, a table with each interpolated fishing position with its corresponding fishing time and landings by each commercial species is obtained and stored in ICATMAR’s database.

### Data visualization and products

**Figure 5.**
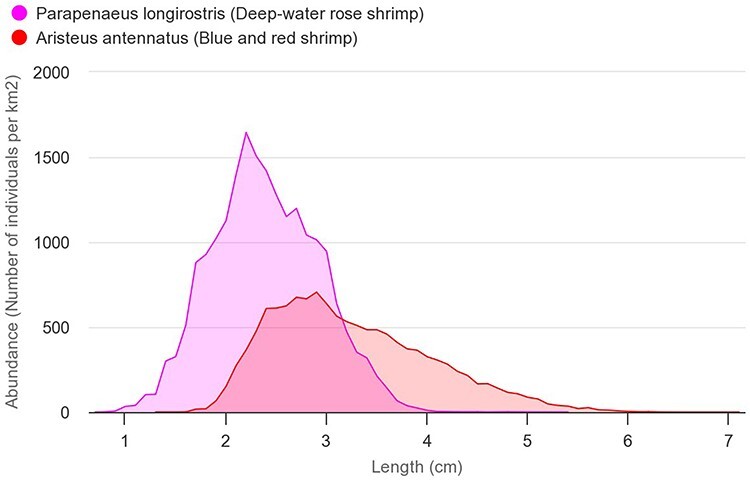
Comparison of the length-frequency distributions of blue and red shrimp (*A. antennatus*—red) and deep-water rose shrimp (*P. longirostris*—pink). The abundances on the chart are the average of all areas and years (number of individuals/km^2^).

**Figure 6.**
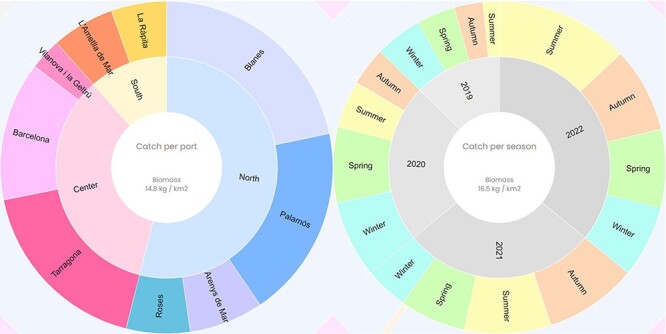
Catch composition per port and season of the deep-water rose shrimp (*P. longirostris*). The value of the central circle is the average of the species’ biomass per port or season (kg/km^2^).

**Figure 7.**
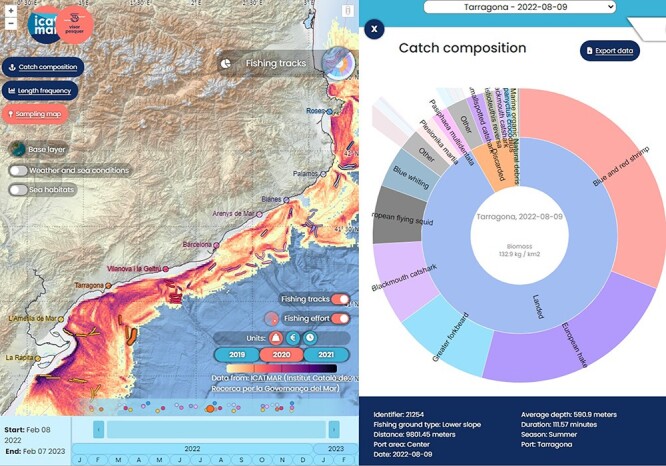
Catch geolocation of a bottom trawling sampling. An additional layer with the biomass extracted for all the fishing fleet (kg/km^2^) is added to the map.

The ICATMAR’s database provides service to several data products. In this work, we present three examples of web-based products: length distribution charts per species (Figure 5), catch composition per port or season (Figure 6) and catch geolocation of bottom trawl surveys (Figure 7). For each product, the data visualization is explained in the following paragraphs.

The data extraction from the database is done following the same process for all the products described in the following sections. A node.js server (20) executes SQL queries to the database to extract the required data for each product. This server could make the petitions on demand, but since the database is only updated weekly, the responses of the query are stored as static files in the data products. The web products are developed with Hypertext Markup Language 5, Cascading Style Sheets, JavaScript, and external libraries.

#### Length distribution charts

This data product visualizes the length distribution charts for each species based on the bottom trawl sampling data. Several species can be displayed together in the same chart through the web interface (Figure 5).

For the length distribution charts, the SQL queries aggregate and average the abundance metrics explained in the data processing section. In mathematical terms, the abundance (number of individuals per kilometer squared) for each species (*x*) and length classes (*y*) is computed as follows:


(3) $$Aggr.\,\, Abundance_{xy}\left(\frac{NIndividuals}{km^2}\right)\;=\;\frac1N\sum_{z=1}^{NS}Abundance_{xyz}$$

where *N* is the number of hauls and *NS* is the number of samples in these hauls. *Abundance* is the number of individuals per kilometre squared for the species *x* and length class *y* in the sample *z*, as explained in the data processing section.

The charts were created using the library Highcharts (21). This library allows a chart to display several graphs at the same time. An interface with a search bar was added to be able to filter by species.

#### Catch composition

This web visualization summarizes the biomass for each species of the bottom trawl sampling for each port and season using pie charts (Figure 6).

To obtain the catch composition per port and season, the SQL queries aggregate and average the biomass for each species. In mathematical terms, the species biomass (kg per km^2^) for each species (*x*) and category (*y*) is computed as follows:


(4) $$Aggr.\,\, Biomass_{xk}{}{\left(\frac{kg}{km^2}\right)}{}={}\frac1N\sum_{z{}={}1}^{NS}Biomass_{xkz}$$

where *N* is the number of hauls and *NS* is the number of samples in these hauls. *Biomass* is the weight in kg per km^2^ of the species *x* and category *k* in the sample *z*, as explained in the data processing section. The query selects the hauls that belong to a port or a season, depending on the type of product.

The pie charts use the library of D3 (22) and are based on the zoomable sunburst pie charts (23). The interactive pie charts have different levels, allowing the user to navigate through them and hover over the partitions to see the aggregated biomass at each level. The web interface allows to compare pie charts, side by side, at species level, i.e. plotting the biomass distribution for certain species. In detail, the port pie charts have four different levels: area (north, central, south), port (a total of nine ports), category (landed, discarded, natural debris, and marine litter) and species biomass. Similarly, the seasonal pie charts have four levels: year, season (spring, summer, autumn, winter), category and species biomass. The biomass is aggregated at the highest levels, e.g. the total biomass of an area/port or year/season including all categories. The user can filter the pie charts by species. This feature allows the user to see the distribution of determined species over different areas or seasons. Data can be downloaded for each pie chart.

#### Catch geolocation

This data product is an interactive map that joins the sampled hauls’ catch composition with the geographic location, time and specific weather and sea conditions. The hauls’ catch composition can be seen together with the trawling fleet activity through fishing effort and landings distribution maps.

The base map shows the GPS tracks for a sampling haul together with geographical and bathymetrical information. Moreover, a side panel displays information on the haul’s catch composition and the sea conditions at the time. Additional layers can be added to the map such as fishing effort, seabed habitat and sea conditions (data models and observations of sea temperature, salinity, chlorophyll, etc.). On the bottom of the application, the dates of the sampled hauls are shown on an interactive calendar (Figure 7).

For each haul, the following information is extracted from the database: catch composition, GPS track, average depth, fishing ground characteristics and duration. The queries to the database for this data product have a similar structure as in the previous product named ‘catch composition’. The main difference is that the biomass data are aggregated per haul.

Maps of fishing effort, biomass and revenue per area (kilometre squared) are added to the visualization, using data from VMS and landings, as explained in the data processing section. These maps are stored as images and used in the interactive web application.

Data about the sea conditions are requested on the fly from the E.U. Copernicus Marine Service Information (CMEMS) through Web Map Service (WMS). These queries provide data from models and satellite observations about sea surface temperature, sea bottom temperature, sea surface temperature anomaly, sea surface velocity (sea current) (24–28), chlorophyll (29), wind (30), wave significant height, and wave period (31). Different CMEMS products are used for the same variables to cover the time period of the data.

The information on the seabed habitats is extracted from the European Marine Observation and Data Network (EMODnet) Seabed Habitats initiative (32). The geographic distribution of the seabed habitats is stored as a static image map. For each haul, detailed information on seabed habitats of the area are extracted using the Web Feature Service (WFS) provided by EMODnet. These WFS requests are done by the client, i.e. every time the user clicks on a haul, the seabed habitat is requested from the WFS using the geographic location of the haul.

The panels and interface were created with Vue.js v3 (33). The map visualization uses the library Openlayers v6 (34). The pie charts are created using the same technologies as in the previous data product.

## Results

### Data ingestion and quality

The monitoring program is ongoing since 2019. The results herein presented include data from 2019–2022, thus a total of 4 years of data. During this period, the onboard sampling program performed a total of 406 hauls, with an average (± SE) of 102 ± 6 hauls per year (Table 1). In total, all the fishing trips combined lasted 1497 h, representing a swept distance and area of 3108 km and 67 km^2^, respectively. From all the collected samples, a total of 535 taxa were identified, 84% of them to species level. In detail, the current dataset comprises approximately 250 000 entries of measured individuals and 36 000 of dissected ones. For each sampling, the whole catch is classified but only a fraction is measured. Therefore, extrapolating the measured individuals to the whole catch of the sampling, the number of sampled individuals is 1 100 000, which represents 24.6 tons of catch. All these data were entered through the data input website, processed and stored in the database.

**Table 1.** Bottom trawling sampling statistics from 2019 to 2022 with 84% of the specimens identified at the species level. SE means Standard Error

Parameters	2019	2020	2021	2022	Total	Annual average ± SE
Number of hauls	116	89	102	99	406	102 ± 6
Trip duration (h)	466	328	349	354	1497	374 ± 31
Swept distance (km)	936.0	699.1	775.5	696.8	3,107.4	776.9 ± 56.1
Swept area (km^2^)	19.9	14.7	16.4	15.6	66.9	16.7 ± 1.1
Number of sampled specimens	317 889	245 858	299 949	236 736	1 100 432	275 108 ± 19 948
Sampled catch (*t*)	7.9	4.8	5.7	6.1	24.6	6.1 ± 0.6
Number of measured specimens	74 527	50 853	60 780	60 758	246 918	61 730 ± 4864
Number of dissected specimens	10 101	7619	8275	10 339	36 334	9084 ± 671
Different identified taxa	435	364	385	364	535	387 ± 17

The external fisheries data registers integrated into the database are approximately 26 million daily landings, 150 000 EU fleet registers and 54 million interpolated VMS points.

**Figure 8.**
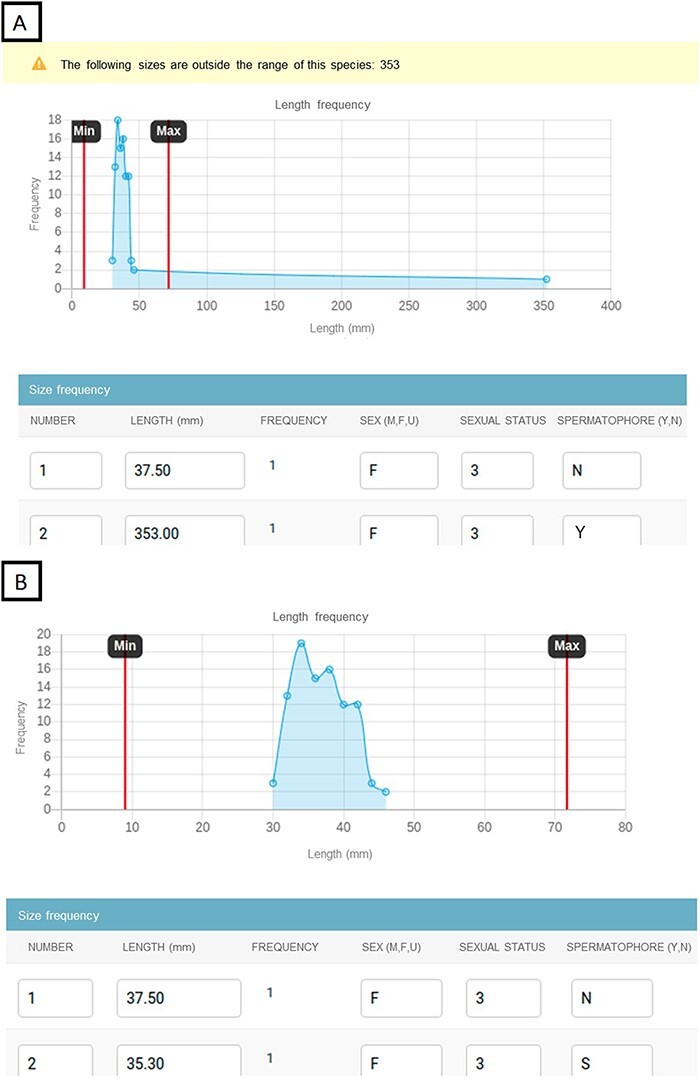
Example of the data input website to prevent incorrect data. The website displays a line chart with the length frequency and species’ length ranges. If some lengths are out of the range, they can be detected in the length frequency chart and the website triggers a warning with the lengths that need to be checked. Chart showing the lengths out of range with a warning detecting the outliers (A). Chart showing the fixed lengths that fall within the range values (B).

Regarding data curation, from 2019 to 2022, the validation workflow of the data input detected and corrected a total of 1681 erroneous data entries from the original sampling forms As an example of defect prevention effectiveness, 189 length outlier values were registered of 33 224 data points during the first 6 months of sampling, when the database and website were still not running. For a period of 6 months once the system was running, 0 length outlier values were registered of 32 141 data points, as the website triggers a warning when a length value is out of the usual range of the species and displays a chart to perform a visual validation (Figure 8).

### Use cases

In this section, two use cases are described. The use cases are examples of specific scientific questions that are of current interest to show the potential of the system presented here. The first use case is related to the appearance of a shrimp species on the Catalan coast, whereas the second one shows the impact of a storm in the marine litter caught with bottom trawls.

#### Warming of the Mediterranean Sea and *Parapenaeus longirostris*

The water temperature of the Mediterranean Sea has been increasing over the past years (35), a fact that is suspected to alter the habitats and distributions of certain species (36, 37). The blue and red shrimp (*Aristeus antennatus*) is extensively commercialized being a main target species for the fishing sector. In the recent years, the deep-water rose shrimp (*Parapenaeus longirostris*) has increased its abundance and presence becoming one of the top commercial species of the study area, probably favored by warmer Mediterranean waters (38). To understand and analyze the presence of both shrimp species and any potential interaction between the species, several questions need to be answered. The system presented in this work can contribute answering some of these questions (Figure 2).

Regarding the sampling data, the catch composition per port shows that the deep-water rose shrimp is mostly fished in the northern and central area of the Catalan coast. The fact that its biomass is much lower in the south, might be related to the shallow depths of the wide continental shelf of the Ebre Delta, which covers most of the southern trawling area. The catch composition per season shows that the deep-water rose shrimp is fished all year round, and the fished biomass increases yearly (Figure 6).

The blue and red shrimp follows a similar distribution in terms of geographic area (Supplementary Figure S2), along with a similar biomass (16.6 kg/km^2^) than the deep-water rose shrimp (14.8 kg/km^2^). Although the catch per port is similar for both species, the sampled hauls of the catch geolocation show that these two species are caught in different locations and do not share the same habitat (Supplementary Figure S3): the deep-water rose shrimp is mainly found in the deeper continental shelf and the upper slope, at depths between 100–400 m, whereas the red and blue shrimp is mainly found in the lower slope (>450 m depth). Nevertheless, when comparing the catch composition per season between 2019 and 2022, it is observed that the deep-water rose shrimp has overtaken the blue and red shrimp in terms of biomass, becoming one of the most important crustacean catches in the Catalan coast (Supplementary Figure S4).

Almost all individuals caught for both species are commercialized and never discarded (Supplementary Figure S5 as an example). The length distribution chart shows that deep-water rose shrimp individuals <2 cm are commercialized, when they should be discarded because their minimum conservation reference size (MCRS) in the Mediterranean is 2 cm (Figure 5). This is an indication that MCRS management measure for the deep-water rose shrimp is not fully implemented by the fishing fleet.

#### Storm Gloria and marine litter in the catch

Between the 20th and 23rd of January 2020, the storm Gloria hit the Catalan coast with waves over 8 m high and rainfall up to 400 mm in a single day, resulting in flooding and significant changes along the Catalan coastline (39). This type of events tends to increase the amount of marine litter in the sea, as mismanaged waste is flushed directly to the sea along with water that has not gone through wastewater treatment plans because of capacity limitations (40). Then, the accidental catch of marine litter may be more abundant after extreme weather events, increasing the probability to damage fishing gears, and even the vessel’s propellers (41, 42). The data collected and its visualization allows the visual analyses of benthic marine litter along the Catalan coast and the understanding of how extreme weather events may affect its presence, abundance and composition. The visualization ‘catch composition’ per season shows that, in spring 2020, the marine litter found was almost double the marine litter found in other seasons (Supplementary Figures S6 and S7). That spring, about 60% of the marine litter fished was disposable wet wipes, which compared to springs from other years was unusual. Marine litter data may provide understanding on waste mismanagement and may be used to implement best management strategies both on land and in the sea (43).

The product catch geolocation allows tracking the accidental catch of marine litter after the storm to understand the evolution of the marine waste on fishing grounds. Inside the application, one can select the hauls after the storm Gloria and compare them with the hauls before and after, in different areas and depths. Barcelona is one of the largest cities in Europe (1.6 million of people; Eurostat) and it is limited by two rivers (Llobregat and Besós). There, 16 hauls were done at an approximate distance of 7 km from the coast and at a depth of around 100 m from 2019 to 2022. The data showed an increase in marine litter in the summer of 2020, just 6 months after the storm. The marine litter collected on that area reached a maximum of 365 kg/km^2^ in a specific haul, whereas the months before and after (∼1 year and a half) the storm, these were around 121 kg/km^2^. At >10 km off the coast and at depths >300 m, other hauls (a total of 25) did not show litter biomasses >48 kg/km^2^, probably indicating that the areas adjacent to the coast are more affected by storms and heavy rains.

## Discussion

This work presents methods for all the process from data collection to web visualization from bottom trawl surveys for best fisheries management practices in the NW Mediterranean Sea. The two use cases presented to illustrate the usability of the data and their visualizations exemplify how the interface can be used for scientific analysis, which could lead to science-based governance. Moreover, the addition of external data such VMS or daily landings makes the system more robust (44) and offers a great potential for a variety of users and goals. The main goal of the data management systems is to be used not only for governance and science but also for the industry, such as the fishing sector, and the society at large. This goal is aligned with the FAIR principles to give access to several stakeholders (7), and follows the Ocean Decade Challenge 9—deliver skills, knowledge and technology to give ‘access to data, information, knowledge and technology across all aspects of ocean science and for all stakeholders’ (45). In addition, the fact that the technologies used for the entire system and the developed web visualization are open-source ensures replicability and contributes to a more robust and reliable scientific landscape (46).

Fisheries management should be based on scientific advice provided by onboard observers and port samplings data, among other information (47–49). However, the most difficult obstacle to efficient monitoring and assessment of marine ecosystems is to obtain records of adequate quantity and quality for a region, period and taxonomic group of interest (50–52). Fishery databases, such as the Clodia database in the Adriatic Sea (53), rely on landed or target species rather than ocean monitoring. This is a problem for fisheries science and ecosystem-based management because environmental traits are important to understand fish stocks (48, 54).

The data presented in ICATMAR’s database and their web visualization is provided by an intense monitoring program, in combination with external fisheries data, to offer a comprehensive and detailed visualization for fisheries’ management. Moreover, as all data pre-processing is routinely done and stored, scientific or administration questions can be quickly answered. These data curation improvement efforts are essential to publish high quality and easily accessible data that can help make better management decisions, similar to what other global marine information systems (e.g. OBIS, Ocean Biodiversity Information System) are implementing elsewhere (55).

Open-access scientific data are still limited. For example, more than half of the studies published in journals such as Nature or Science over the past 10 years still lack key data that would allow for re-analysis or meta-analysis (56). Research and management in natural resource science increasingly relies on very large datasets gathered from multiple sources (57), such as the web visualization presented in this study. Web visualization is a way to share science globally while lowering the costs of data collection, which may be costly and time consuming. Moreover, web visualization facilitates the re-analyzes of the data, confirms results, spots errors and gains understanding (58) on fisheries science.

As far as the authors know, there are few open-data portals displaying similar information. For example, the Irish Groundfish Survey, which is part of the Ireland’s data collection scheme (44), shows similar information for 19 target species (https://shiny.marine.ie/igfs/). However, as opposed to the web visualization presented here, the data do not integrate information on discards or marine litter and it only shows the information per species, missing the holistic perspective of the ecosystem approach that fisheries management should seek (48, 54).

The fishing industry is one of the most interested stakeholders to get and understand scientific fisheries data. This understanding is of great importance when moving towards co-management strategies, where the different stakeholders involved need to be engaged at each stage of the process (47). In order to achieve fisheries sustainability in biological, social and economic aspects, co-management encourages direct users, governments and other actors to jointly manage the resources of the fisheries (59). In the NW Mediterranean, Spain promoted the Plan Castelló (1961–1966), an officially recognized co-management system that demonstrated a recovery of the fishing stocks and an increase in the catch for the main commercial species (60). Currently, co-management has gained importance globally on the premise that fishers’ participation in resource regimes increases compliance, reduces transaction costs and raises authority over decisions (61). To better implement co-management strategies, the web visualization here presented may be used as a tool to facilitate comprehension of the scientific data to the fishing industry easing the dialogue between science and fisheries.

Several challenges from the Ocean Decade highlight the importance for society to understand and value the ocean and how it relates to sustainability development and human well-being (45). In fact, the Ocean Decade has six different societal outcomes, including safety, sustainability and prediction among others, which require actions by the society, administrations, or by key stakeholders to be fully achieved (62). The need to connect society and ocean research (63) may be ameliorated by using formal and informal educational strategies, as well as tools for raising awareness (64). Then, the web visualization may be used not only as an easily accessible and interactive tool for awareness but also, it can be helpful for educational goals. Therefore, the web visualization can be used as an educational tool to engage students in marine and fisheries sciences because teaching must involve students in real-world situations, providing them the chance to develop theories and evaluate those theories based on empirical data (65). So far, the bottom trawl sampling data are collected in ICATMAR’s database, processed and visualized, but to reach other relevant stakeholders, small-scale fisheries, purse seine and recreational fishing samplings will be added into the data management systems as future improvements.

Fisheries databases include high volumes of data, but static reports are limited to targeted species and specific metrics. Thus, the end-to-end system, from data collection to web visualization, presented in this study allows all stakeholders to navigate the data through a user-friendly tool to gain engagement and promote ocean sustainability. The visualizations proposed here are the first step towards an open data visualization application of fishing catches on the Catalan coast.

## Supplementary Material

baad067_Supp
