# Supplementary material for: Fisheries data management systems in the NW Mediterranean: from data collection to web visualization
Source: Database (Oxford). 2023 Oct 20;2023:baad067. doi: 10.1093/database/baad067 (PMC10590195; doi:10.1093/database/baad067)
Supplement: baad067_Supp [file baad067_supp.zip › suppl_data/20230731_Supplementary_material_revision_NonTracked.docx]

# Supplementary Material


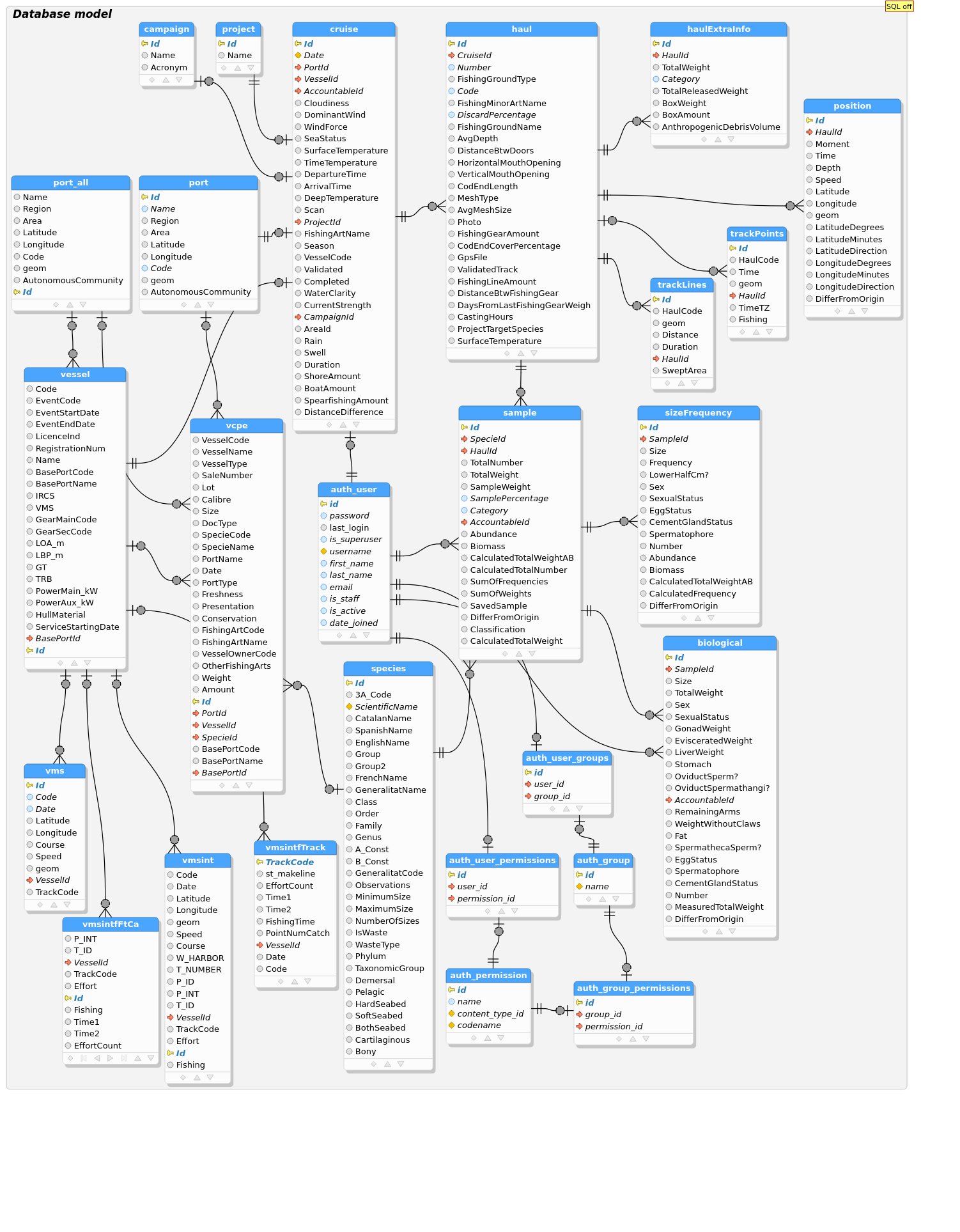


Figure S1. ICATMAR’s database model.


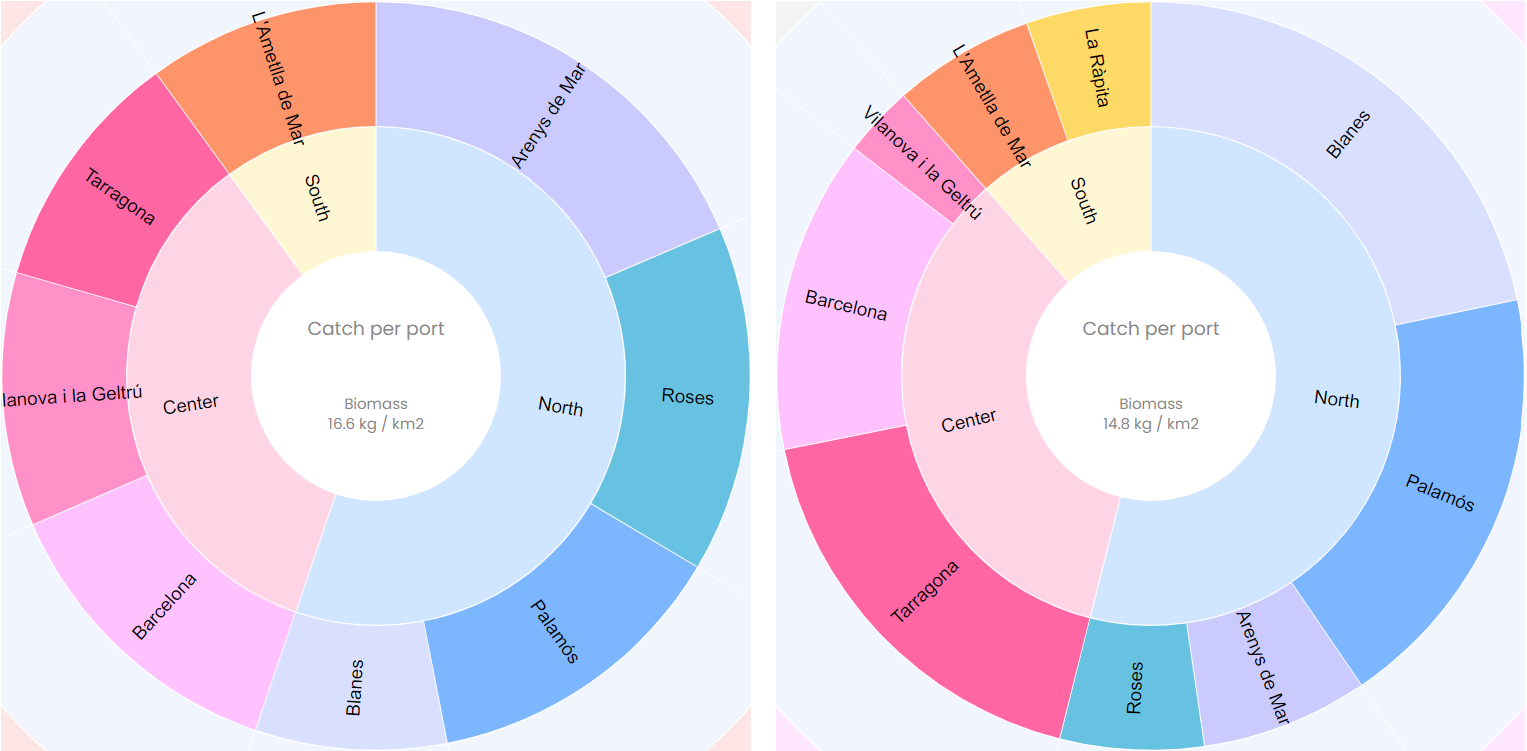


Figure S2. Comparison of catch distribution per port of blue-red shrimp (*Aristeus antennatus* - left) and deep-water rose shrimp (*Parapenaeus longirostris* - right).


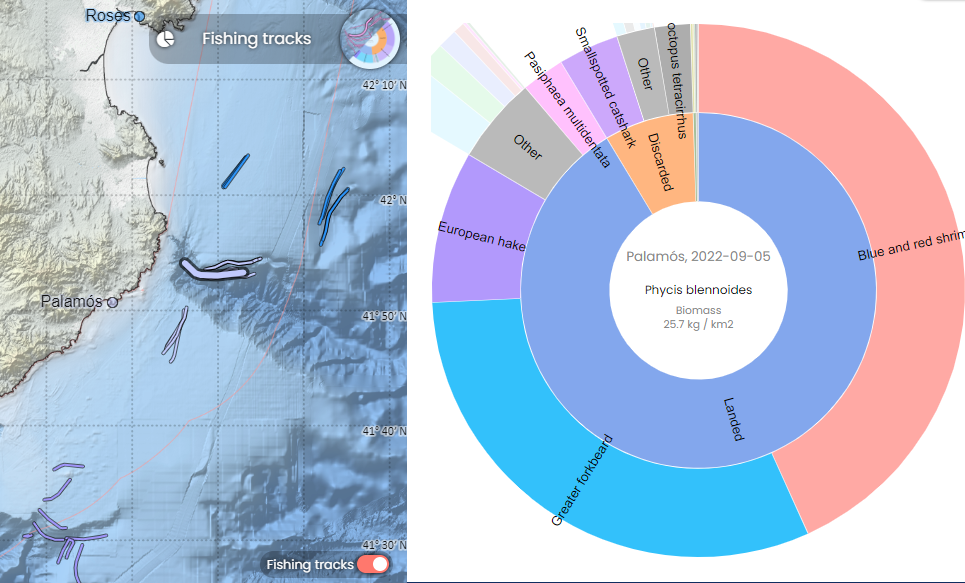

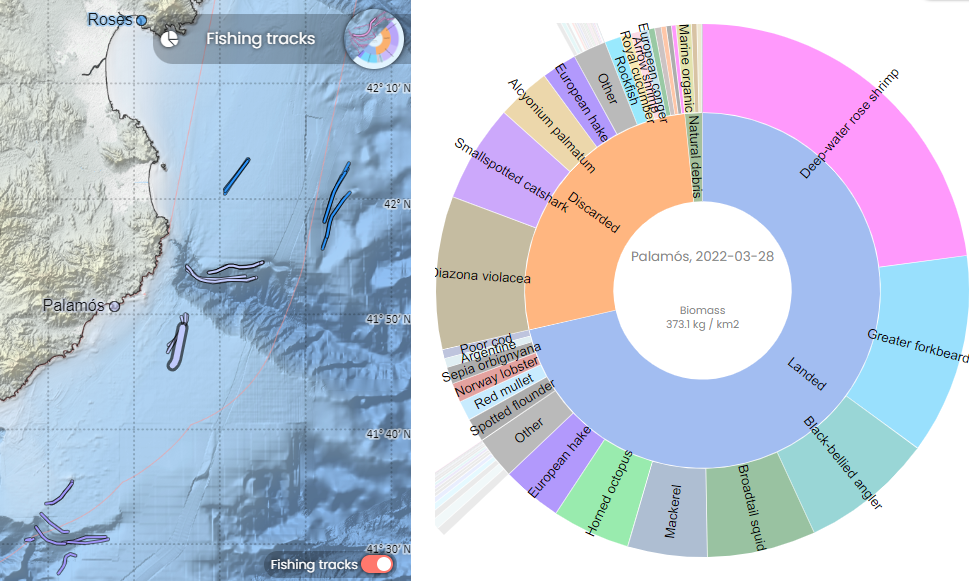


Figure S3. Two geolocated catch compositions. Both catch compositions show that deep-water rose shrimp (*Parapenaeus longirostris*) and blue and red shrimp (*Aristeus antennatus*) are not distributed in the same fishing grounds.


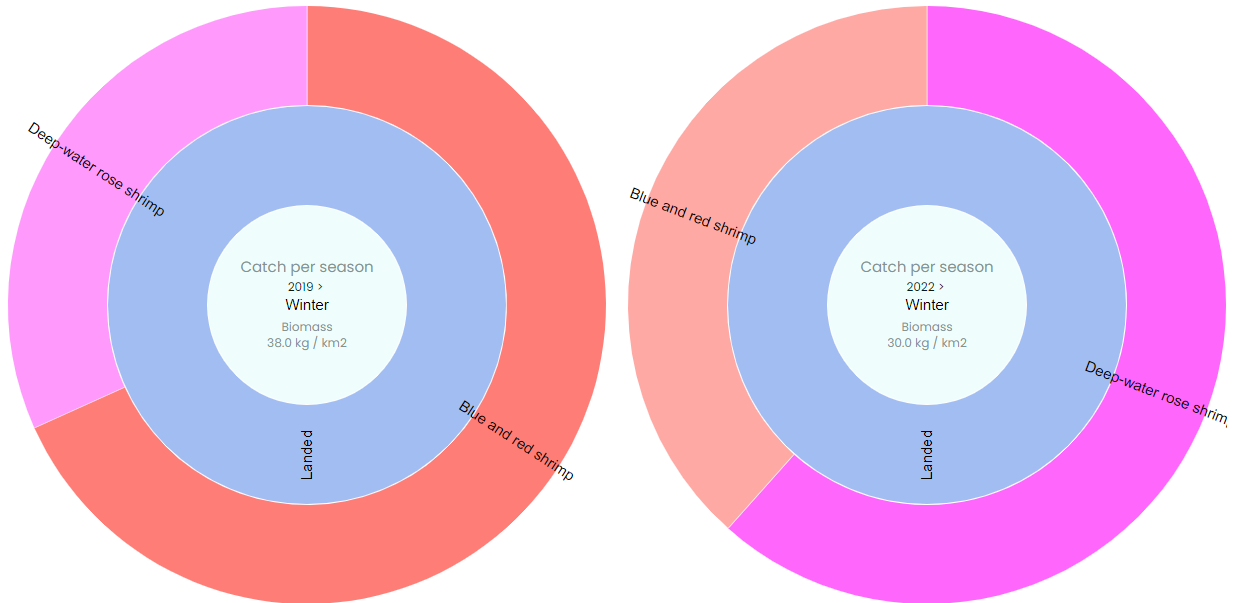


Figure S4. Catch composition per season of deep-water rose shrimp (*Parapenaeus longirostris*) and blue and red shrimp (*Aristeus antennatus*), comparing the winter of 2019 (left) with the winter of 2022 (right).


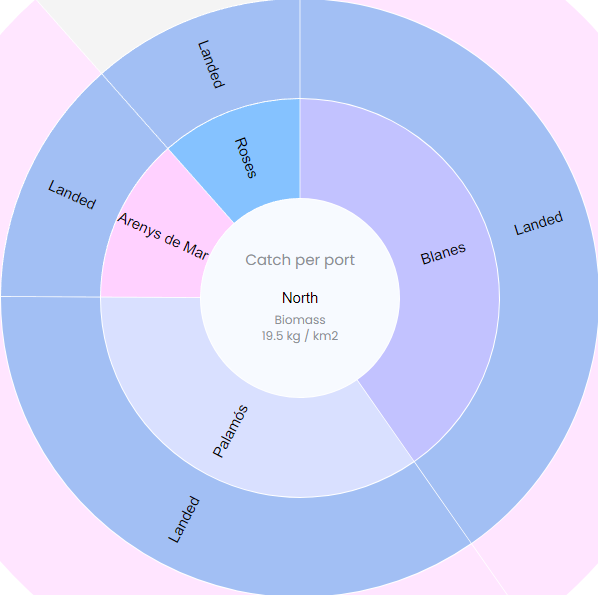


Figure S5. Catch composition of the northern Catalan coast of deep-water rose shrimp (*Parapenaeus longirostris*). Note that none of these individuals are discarded.


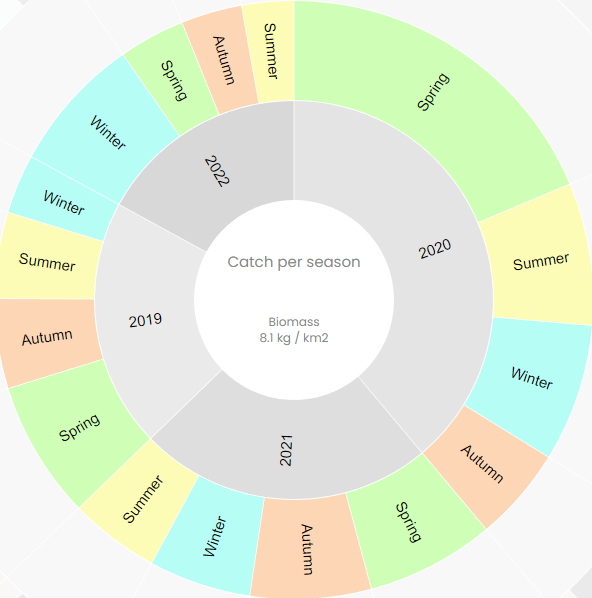


Figure S6. Catch composition per season of marine litter. Note how in the spring of 2020 an unusual increase of marine litter occurred, probably related to an unusual storm period.


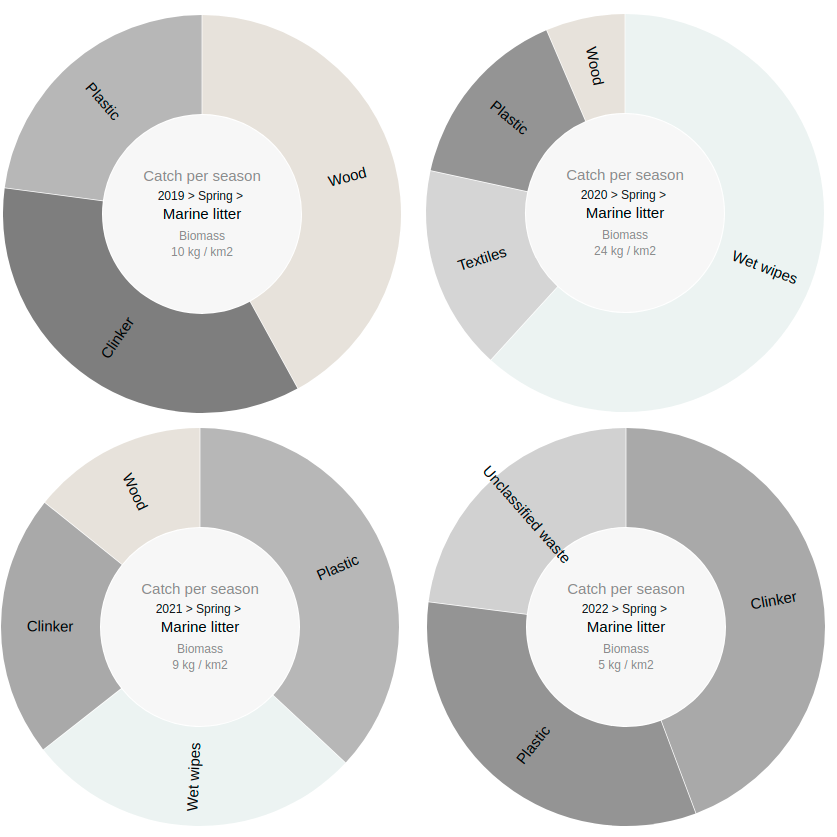


Figure S7. Spring’s marine litter composition of years 2019 and 2022. Biomass per km^2^ was more than double in the spring of 2020 (24 kg / km^2^) and mostly wet wipes were present in the sample in comparison with other years.
