# Supplementary material for: Fisheries data management systems in the NW Mediterranean: from data collection to web visualization
Source: Database (Oxford). 2023 Oct 20;2023:baad067. doi: 10.1093/database/baad067 (PMC10590195; doi:10.1093/database/baad067)
Supplement: baad067_Supp [file baad067_supp.zip › suppl_data/ORCIDs IS.docx]

**ORCIDs**

**Jordi Ribera-Altimir**

0000-0002-8232-3334

**Gerard Llorach**

0000-0001-6744-291X

**Joan Sala-Coromina**

0000-0003-1221-7648

**Joan B. Company**

0000-0002-5878-7155

**Eve Galimany**

0000-0003-1493-2944
